# The relationship between intimate partner violence and child malnutrition: a retrospective study in 29 sub-Saharan African countries

**DOI:** 10.3389/fpubh.2023.1231913

**Published:** 2024-01-05

**Authors:** Kunhong Lin, Pengxiang Zhou, Mengyuan Liu, Botian Chen, Zibei Zhou, Yijia Zhang, Ying Zhou, Yanan Jiang, Shuyun Bao, Dijia Chen, Yu Zhu, Yan Xing

**Affiliations:** ^1^Department of Pediatrics, Peking University Third Hospital, Beijing, China; ^2^Department of Pharmacy, Peking University Third Hospital, Beijing, China; ^3^Institute for Drug Evaluation, Peking University Health Science Center, Beijing, China; ^4^School of Public Health, Peking University Health Science Center, Beijing, China; ^5^Peking University Health Science Center, Beijing, China

**Keywords:** intimate partner violence (IPV), sub-Saharan Africa (SSA), stunting, wasting, underweight

## Abstract

**Introduction and background:**

Intimate partner violence (IPV) and child malnutrition are global public health issues. Assessing the association between IPV and child anthropometric failures (stunting, underweight, and wasting) in 29 Sub-Saharan African (SSA) countries can provide significant global health solutions. Some studies have found an association between IPV against women and child malnutrition, but the conclusions are inconsistent. The physical and psychological conditions, living environment, and rights of the mother may be involved.

**Methods:**

We collected and analyzed the Demographic and Health Surveys data (2010–2021) of 29 SSA countries. The main exposure variables were various types of IPV, classified as physical, sexual, and emotional violence. The outcome was the child’s development index, which can be roughly divided into stunting, wasting, and underweight. An adjusted binary logistic regression model was used to test the relationship between IPV and children’s nutritional status.

**Results:**

A total of 186,138 children under 5 years of age were included in the analysis; 50,113 (27.1%) of the children were stunted, 11,329 (6.1%) were wasted, and 39,459 (21.3%) were underweight in all regions. The child’s gender, age, duration of breastfeeding, complementary feeding, and vitamin A supplements intake in the past 6 months were associated with their nutritional status (*p* < 0.001). Sexual violence was the strongest factor associated with stunting, which remained statistically significant after controlling all variables (AOR = 1.11; 95% CI: 1.02, 1.21; *p* = 0.012). We also found a small negative association between wasting and IPV. For underweight, there were no associations with IPV after controlling for all variables (*p* > 0.05).

**Conclusion:**

IPV is positively associated with child stunting in SSA countries. Sexual violence showed a strong positive correlation with stunting. Wasting was unexpectedly negatively associated with IPV. There was no clear correlation between underweight and violence.

## 1 Introduction

Violence against women, a major public health issue and a violation of women’s human rights, has a far-reaching influence worldwide. On the basis of the World Health Organization (WHO), the most widespread form is intimate partner violence (IPV), referring to the behavior by an intimate partner or ex-partner that results in physical, sexual, and mental harm, including physical aggression, sexual coercion, psychological abuse, and controlling acts. Globally, nearly one-third (27%) of women aged from 15 to 49 who have been in a relationship declared that they have experienced various forms of physical and/or sexual violence from an intimate partner (1). A systematic review and meta-analysis in Sub-Saharan Africa (SSA) demonstrated that the prevalence of IPV among women was 44% during their lifetime, in which emotional violence accounted for 29.40%, physical violence accounted for 25.87%, and sexual violence accounted for 18.75% (2). Violence against women can negatively affect women’s physical, mental and reproductive health-specially infertility, miscarriages, stillbirths, and induced abortions (3–5). These may be related factors that affect the growth and development of children after birth. Additionally, it may increase the risk of acquiring human immunodeficiency virus (HIV), breast and gynecologic cancers, and even the global prevalence of intimate partner homicide and under-5 mortality (6–8).

Malnutrition also has a high prevalence in SSA, with one-third of all undernourished children living there (9). Stunting, underweight, and wasting are indicators of undernutrition. Studies on child malnutrition conducted in SSA countries have identified potential causal factors, mainly including (1) maternal-related factors: low mother’s education level, mother’s age (<20 years), and low mother’s BMI (<18.5 kg/m^2^); (2) child-related factors: increasing child’s age, children’s gender (boy), low birth weight, and prolonged duration of breastfeeding (>12 months); and (3) household factors: wealth index (poor), source of drinking water (unimproved), no access to media, and residence (rural) (10–13). Researchers studying children’s developmental and nutritional outcomes are increasingly recognizing the importance of IPV. Previous studies have reported the associations of IPV with child anthropometric indicators. A study in Peru found that there was no evidence of an association between malnutrition and other kinds of violence inflicted except physical violence by a partner (14). Stunting was found to be positively associated with physical or sexual violence, and there is a small negative association between wasting and IPV in the study from 42 demographic and health surveys (15). Moreover, a study in Nigeria found children whose mothers experienced IPV were less likely to be underweight (16). Due to the inconsistency in the scope and inclusion criteria of the above studies, the conclusions remain inconsistent.

To the best of our knowledge, there is a lack of literature that clearly illustrates the relationship between IPV against women and the nutritional status of children under 5 years of age in SSA countries. This cross-sectional study aimed to explore the associations of exposure to the various types of IPV with childhood malnutrition status via nationally representative data from Demographic and Health Surveys (DHS) from 29 SSA countries.

## 2 Methods

### 2.1 Study design

This cross-sectional study examined the association between the nutritional status of children under 5 years of age and maternal IPV in SSA in the following ways: (1) in 29 Sub-Saharan countries, we described the incidence of the nutritional status of children by countries and regions (East Africa, Southern Africa, West Africa, and Central Africa), which provides a picture of their prevalence. (2) We examined the differences in outcome event rates due to exposure to various factors, including the independent variable (IPV) and the covariates (mainly including family factors, child factors, and maternal-related factors). (3) We explored the association between women’s exposure to various types of IPV (physical violence, sexual violence, and emotional violence) and the nutritional status of their children, and introduced the combination of all types of violence and time (in the last 12 months) of each type of violence to study the impact of violence on children’s nutritional status in different situations.

### 2.2 Data source

This study analyzed secondary data from the DHS. DHS sample designs were two-stage probability samples drawn from an existing sample frame, generally the most recent census frame (17). Each country’s survey consisted of different datasets including men, women, children, birth, and household datasets. These latest datasets collected in 29 SSA countries from 2010 to 2021 were appended together to investigate the association between the nutritional status of children under 5 years of age and maternal IPV. Other countries with missing research data, not participating in public surveys, and outdated data were excluded. In this study, we combined the individual and children recode datasets. The study participants recruited were children under 5 years of age. We included their mother’s exposure to IPV as an important factor in the analysis. In addition, the nutritional status of children under 5 years of age in these families was classified based on the z-values of weight and height in the secondary data. Our study followed the Strengthening the Reporting of Observational Studies in Epidemiology (STROBE) reporting guideline. The STROBE list consisting of 22 items describes the writing points of article elements such as title, abstract, background, purpose, research design, statistical methods, results, and discussion (Supplementary File).

### 2.3 Variables

The coding plan of the selected study variables is shown in Supplementary File 1. Using WHO growth criteria as a reference, stunting, underweight, and wasting were defined as Z-scores less than −2 standard deviations (SD) from the median height for age (HAZ), weight for age (WAZ), and weight for height (WHZ), respectively. In other words, “stunting” means HAZ < −2 and “no stunting” means HAZ ≥ −2; “underweight” means WAZ < −2 and “no underweight” means WAZ ≥ −2; and “wasting” means WHZ < −2 and “no wasting” means WHZ ≥ −2 (18). The main exposure variables were types of IPV, which were classified as physical violence, sexual violence, and emotional violence. Among them, physical violence is based on these conditions: ever been pushed, shaken, or had something thrown by the husband/partner; ever been slapped by the husband/partner; ever been punched with a fist or hit by something harmful by the husband/partner; ever been kicked or dragged by the husband/partner; ever been strangled or burnt by the husband/partner; ever being threatened with knife/gun or other weapons by the husband/partner; ever experienced CS physical violence by the husband/partner; and ever had arm twisted or hair pulled by the husband/partner. Sexual violence is defined as ever being physically forced into unwanted sex by husband/partner, forced into other unwanted sexual acts by husband/partner, or physically forced to perform sexual acts respondent did not want to. Additionally, emotional violence is considered as ever being humiliated by the husband/partner, threatened with harm by the husband/partner, insulted, or made to feel bad by the husband/partner. We also created the presence or absence of IPV in the past 12 months by time.

Moreover, several covariates, such as child’s gender, child’s age in months, duration of breastfeeding, complementary feeding, and vitamin A supplements intake in the last 6 months classified as child’s factors, wealth index and type of place of residence classified as household factors, maternal education, marital status, maternal BMI, and hemoglobin level classified as maternal-related factors, may have influenced the results. Factors that have been reported in the literature were identified as covariates for assessing child nutritional status include the child’s age in months, child’s gender, number of birth orders, birth weight, diarrhea in the past 2 weeks, mother’s age group, mother’s highest level of education, place of residence, mother’s BMI, and wealth quintile (9, 19). These factors are associated with stunting, underweight, and wasting conditions in children under 5 years of age. Therefore, relevant confounding factors were considered in this study and corrected to obtain more reliable results.

### 2.4 Statistical analysis

**Figure 1:**
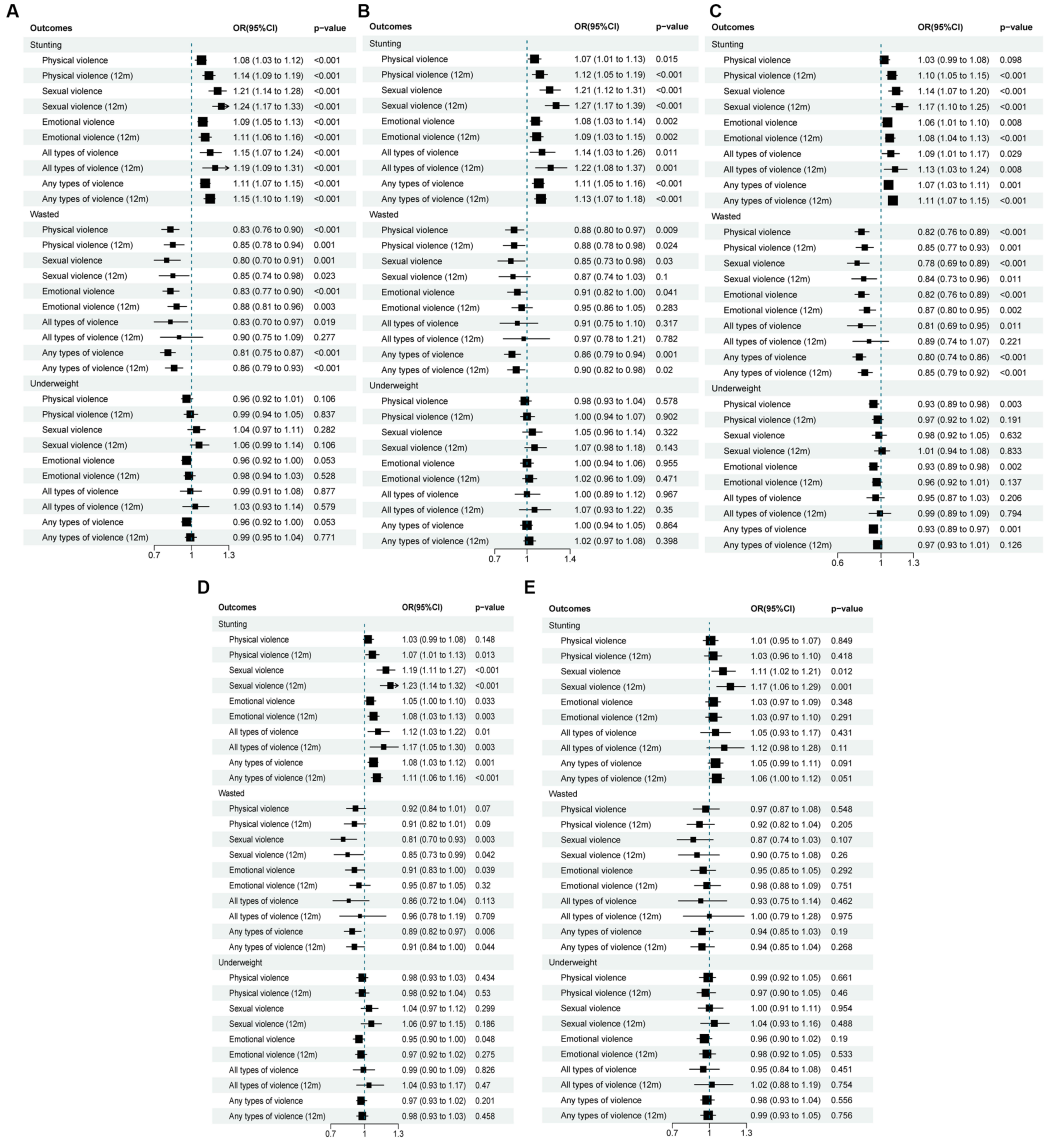
Odds ratio with 95% CI for the association between different forms of IPV and child malnutrition **(A)** Model 1, crude model. **(B)** Model 2, adjusted for child factors: child’s gender, child’s age in months, duration of breast-feeding, complementary feeding, vitamin A in last 6 months. **(C)** Model 3, adjusted for household factors: wealth index, type of place of residence. **(D)** Model 4, adjusted for maternal-related factors: maternal education, marital status, BMI, hemoglobin level. **(E)** Model 5, full model including all covariates.

IPV and child nutrition status were statistically described for each country to reveal the prevalence. The differences between the incidence of outcome events caused by various independent variables and covariates were tested by Pearson’s chi-square test. An adjusted binary logistic regression model was used to examine the relationship between maternal IPV experience and children’s nutritional status (stunting, underweight, and wasting) (20). Four weighted multivariable logistic regression models for each measure of child undernutrition were used to correct the odds ratio (OR). The first model controlled for child-related factors, namely the child’s gender, child’s age in months, duration of breastfeeding, complementary feeding, and vitamin A supplements intake in the last 6 months. The second model controlled for variables related to household factors: metropolitan status and the household wealth index. The third model controlled for maternal-related factors: maternal education, marital status, BMI, and hemoglobin level. The final model included all variables representing the three domains (child, maternal, and household factors) and measures of IPV. This approach tested the impact of IPV on child malnutrition after eliminating corresponding relevant factors to obtain the adjusted odds ratio. The regression results were presented as the estimated adjusted odds ratio (AOR) with 95% confidence intervals (CI). The significance level of regression analysis was set at a *p*-value of <0.05. All statistical analyses were performed using STATA version 15.1. Moreover, we used R software to draw the forest plots, which describe the effects of various forms of violence and developmental indicators (Figure 1).

## 3 Results

### 3.1 Participants’ distribution and the prevalence of outcomes

**Figure 2:**
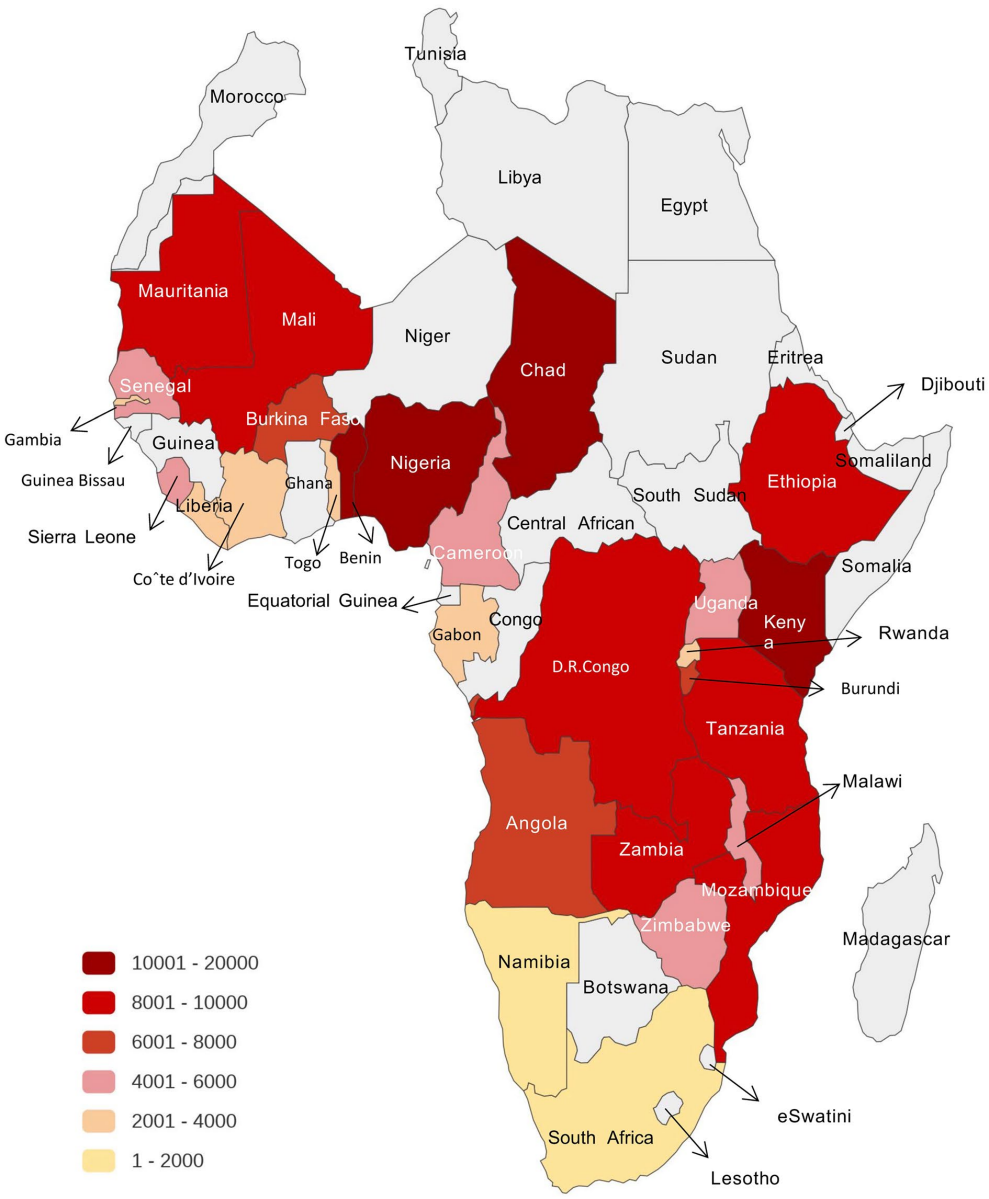
The distribution of participants in each study country from Sub-Sahara Africa different colors represent different population densities. Countries that do not display color are excluded from the scope of study.

In the study, a total of 186,138 children under the age of 5 were included, of which 80,864 (43.4%) were from East Africa, 2,857 (1.5%) from Southern Africa, 70,216 (37.7%) from West Africa, and 32,201 (17.3%) from Central Africa. Figure 2 shows the distribution of participants in each study country. Different colors represent different population densities. Countries that do not display color are excluded from the scope of the study. In East Africa, the prevalence of stunting is 27.8%. In addition, the incidence of stunting in children is 19.0% in southern Africa and 23.8% in West Africa, while the incidence of stunting in Central Africa is the highest, i.e., 33.2%. Central Africa also had the highest incidence of underweight (25.9%). The prevalence of stunting is 47.4%, the underweight rate is 35.1% in Burundi, and this is the highest among countries in the research. The incidence of wasting is relatively low. More importantly, 50,113 (27.1%) of the children were stunted, 11,329 (6.1%) were wasted, and 39,459 (21.3%) were underweight in all regions (Table 1).

**Table 1:** Distribution of study participants and the prevalence of outcomes.

Country (*n* = 186,138)	Prevalence of stunted (%)	Prevalence of wasted (%)	Prevalence of underweight (%)
**East Africa (*n* = 80,864)**	27.8	5.0	19.0
Burundi (*n* = 6,002)	47.4	4.1	35.1
Comoros (*n* = 2,383)	24.1	10.3	18.4
Ethiopia (*n* = 8,844)	31.4	10.9	30.5
Kenya (*n* = 18,630)	21.3	5.2	17.3
Malawi (*n* = 5,109)	28.1	2.6	15.8
Mozambique (*n* = 9,301)	33.4	4.4	17.1
Rwanda (*n* = 3,784)	26.5	1.1	10.4
Tanzania (*n* = 8,903)	26.9	4.4	18.0
Uganda (*n* = 4,396)	22.6	3.4	13.3
Zambia (*n* = 8,623)	27.8	3.5	15.9
Zimbabwe (*n* = 4,889)	19.7	3.4	10.8
**Southern Africa (*n* = 2,857)**	19.0	5.7	14.0
Namibia (*n* = 1,789)	18.6	7.8	17.7
South Africa (*n* = 1,068)	19.7	2.2	7.8
**West Africa (*n* = 70,216)**	23.8	6.8	22.3
Burkina Faso (*n* = 6,523)	29.4	13.7	29.7
Benin (*n* = 11,904)	25.5	4.4	21.1
Coˆte d’Ivoire (*n* = 3,202)	23.7	6.3	19.2
Gambia (*n* = 3,797)	13.0	4.8	16.9
Liberia (*n* = 2,428)	25.5	3.3	15.5
Mali (*n* = 8,495)	22.0	9.0	23.3
Mauritania (*n* = 9,786)	20.7	6.1	21.5
Nigeria (*n* = 11,313)	30.9	5.8	25.7
Sierra Leone (n = 4,099)	24.6	4.8	18.3
Senegal (*n* = 5,486)	14.5	8.8	20.6
Togo (*n* = 3,183)	22.7	6.5	21.1
**Central Africa (*n* = 32,201)**	33.2	7.3	25.9
Angola (*n* = 6,317)	31.4	4.5	22.9
DR. Congo (*n* = 8,035)	38.6	6.9	27.7
Cameroon (*n* = 4,430)	23.5	3.7	12.5
Gabon (*n* = 3,348)	19.5	3.5	10.8
Chad (*n* = 10,071)	38.9	12.3	37.4
All regions (*n* = 186,138)	27.1	6.1	21.3

### 3.2 Distribution of sociodemographic characteristics

The distribution of sociodemographic characteristics of the study population by children’s undernutrition status in SSA is displayed in Table 2. Nearly 40.0% of the women had no education at all, and only 3.0% had college or above education. In total, 128,268 (68.9%) subjects resided in rural areas, and about 47.1% of them lived in poverty. Among the children, 93,828 (50.4%) were boys, 39,894 (21.4%) were infants (0–11 months), 75,450 (40.5%) were toddlers (12–35 months), and 70,794 (38.0%) were of pre-school age (36–59 months). A total of 28.6% of women had experienced physical violence in their lives. A total of 10.0% of women had been subjected to sexual violence by their partners, and 28.5% of women suffered from emotional violence. A total of 5.8% of women had experienced all three types of violence, and 40.3% had experienced any type of violence.

**Table 2:** Distribution of sociodemographic characteristics of the population by children’s undernutrition status in SSA countries.

Factors	Frequency (%)	Stunting			Wasting			Underweight		
		Yes (%)	No (%)	*p*-values	Yes (%)	No (%)	*p*-values	Yes (%)	No (%)	*p*-values
**Maternal-related factors**										
Maternal education				<0.001			<0.001			<0.001
No education	74,147 (39.84)	47.09	36.76		55.26	38.84		52.54	36.04	
Primary	66,309 (35.62)	37.15	35.26		27.52	36.15		32.47	36.66	
Secondary	40,187 (21.59)	14.83	24.25		15.73	21.98		13.93	23.81	
College or higher	5,485 (2.95)	0.93	3.72		1.50	3.04		1.06	3.48	
Missing	10 (0.01)	0.00	0.01		0.01	0.01		0.00	0.01	
Marital status				<0.001			<0.001			<0.001
Never married	35,705 (19.19)	19.01	19.32		13.97	19.52		16.19	20.06	
Married	138,516 (74.42)	73.87	74.55		80.19	74.04		77.37	73.55	
Widowed/Divorced/Separated	11,915 (6.40)	7.13	6.14		5.84	6.43		6.44	6.39	
Missing	2 (0.00)	0.00	0.00		0.00	0.00		0.00	0.00	
Maternal BMI				<0.001			<0.001			0.001
<18.5	13,768 (7.40)	10.14	6.36		16.16	6.83		13.47	5.73	
18.5–24.9	93,159 (50.05)	56.20	47.83		50.21	50.04		53.97	49.05	
≥25.0	33,541 (18.02)	12.29	20.19		10.87	18.48		10.06	20.21	
Missing	45,670 (24.54)	21.38	25.62		22.76	24.65		22.51	25.01	
Maternal anemia level				<0.001			<0.001			<0.001
No	68,045 (36.56)	36.09	36.87		31.09	36.91		32.89	37.68	
Yes	58,359 (31.35)	34.13	30.36		31.36	31.35		33.07	30.92	
Missing	59,734 (32.09)	29.78	32.77		37.55	31.74		34.04	31.40	
**Household factors**										
Residence				<0.001			<0.001			<0.001
Urban	57,870 (31.09)	22.45	34.32		26.10	31.41		22.87	33.34	
Rural	128,268 (68.91)	77.55	65.68		73.90	68.59		77.13	66.66	
Wealth index				<0.001			<0.001			<0.001
Poor	87,624 (47.07)	56.28	43.53		53.02	46.69		56.25	44.47	
Middle	36,882 (19.81)	20.25	19.69		19.04	19.86		19.62	19.90	
Rich	61,632 (33.11)	23.48	36.78		27.94	33.44		24.13	35.63	
**Child’s factors**										
Child’s gender				<0.001			<0.001			<0.001
Male	93,828 (50.41)	52.68	49.56		55.41	50.08		51.52	50.10	
Female	92,310 (49.59)	47.32	50.44		44.59	49.92		48.48	49.90	
Child’s age, months				<0.001			<0.001			<0.001
0–11	39,894 (21.43)	9.21	26.12		18.12	21.65		10.79	24.45	
12–35	75,450 (40.53)	47.52	37.98		55.50	39.56		52.03	37.46	
36–59	70,794 (38.03)	43.27	35.90		26.37	38.79		37.18	38.09	
Duration of breast-feeding				<0.001			<0.001			<0.001
Ever breastfed, not currently	99,154 (53.27)	60.92	50.25		43.46	53.90		55.64	52.47	
Never breastfed	5,072 (2.72)	2.80	2.65		2.91	2.71		2.73	2.68	
Still breastfeeding	65,642 (35.27)	29.08	37.74		46.40	34.54		34.47	35.64	
Missing	16,270 (8.74)	7.20	9.36		7.23	8.85		7.16	9.21	
Complementary feeding				<0.001			<0.001			<0.001
No	61,287 (52.47)	28.81	34.45		35.02	32.79		29.71	33.80	
Yes	55,525 (47.53)	32.45	28.94		35.10	29.49		32.26	29.25	
Missing	69,326 (37.24)	38.73	36.61		29.89	37.72		38.03	36.95	
Vitamin A in last 6 months				0.001			<0.001			<0.001
No	75,851 (40.75)	39.84	40.95		43.27	40.59		40.60	40.66	
Yes	102,461 (55.05)	55.51	55.04		53.29	55.16		54.97	55.22	
Missing	7,826 (4.20)	4.65	4.01		3.44	4.25		4.43	4.12	
**Independent variables**										
Any physical violence				<0.001			<0.001			0.095
No	61,929 (71.36)	70.06	71.80		75.00	71.15		71.84	71.20	
Yes	24,853 (28.64)	29.94	28.20		25.00	28.85		28.16	28.80	
Any sexual violence				<0.001			<0.001			0.013
No	78,102 (90.02)	88.52	90.57		91.95	89.90		89.51	90.14	
Yes	8,659 (9.98)	11.48	9.43		8.05	10.10		10.49	9.86	
Any emotional violence				<0.001			<0.001			0.044
No	62,039 (71.49)	70.09	71.97		74.84	71.29		72.08	71.31	
Yes	24,747 (28.51)	29.91	28.03		25.16	28.71		27.92	28.69	
All types of violence^a^				<0.001			0.006			0.390
No	81,728 (94.20)	93.44	94.48		95.10	94.15		94.06	94.23	
Yes	5,033 (5.80)	6.56	5.52		4.90	5.85		5.94	5.77	
Any types of violence^b^				<0.001			<0.001			0.049
No	51,786 (59.66)	57.67	60.35		64.77	59.36		60.28	59.46	
Yes	35,011 (40.34)	42.33	39.65		35.23	40.64		39.72	40.54	

^a^Ever experienced physical, sexual and emotional violence by partner. ^b^Ever experienced physical, sexual or emotional violence by partner.

For those women who were illiterate, divorced/widowed, underweight, anemic, poor, and living in rural areas, the possibility of malnutrition of their children is higher. In addition, the child’s gender, age, duration of breastfeeding, complementary feeding, and vitamin A supplements intake in the past 6 months were associated with their nutritional status (*p* < 0.001). Children whose mothers had suffered IPV were more likely to be stunted but less likely to be underweight (*p* = 0.049). Only women who had experienced sexual violence had a statistically significant difference in child underweight. However, there were conditions that did not show a statistical difference between physical violence and an underweight child (*p* = 0.095) between all forms of violence and an underweight child (*p* = 0.390).

### 3.3 Association between IPV and the malnutrition index

Figure 1 presents unadjusted and adjusted OR with 95% CI for the association between IPV and childhood malnutrition status. For stunting, in the crude model (Figure 1A: Model 1), women who were identified as having experienced any physical violence were 8.0% more likely to have a stunted child than women who had not experienced physical violence (OR = 1.08; 95% CI: 1.03, 1.12). After controlling for differences in child characteristics (Figure 1B: Model 2), this association remained (AOR = 1.07; 95% CI: 1.01, 1.13). However, the inclusion of household-related (Figure 1C: Model 3) and maternal-related (Figure 1D: Model 4) characteristics completely weakened the correlation between maternal IPV and childhood stunting (*p* > 0.05). In addition, physical violence in the past 12 months increased the correlation between maternal IPV and childhood stunting (OR = 1.14; 95% CI: 1.09, 1.19). Mothers who had experienced sexual violence had a 21.0% greater likelihood of having a child with developmental delay (OR = 1.21; 95% CI: 1.14, 1.28), which remained statistically significant after controlling for relevant factors or even all variables (Figure 1E: Model 5; AOR = 1.11; 95% CI: 1.02, 1.21). Similarly, sexual violence in the last 12 months significantly increased the likelihood of stunting (OR = 1.24; 95% CI: 1.17, 1.33). The risk of stunting was 1.1 times higher (95% CI: 1.05, 1.13) for children whose mothers experienced emotional violence than for those whose mothers did not experience emotional violence, and the association did not change with the adjustment of mother, child, and household factors (Figures 1B–D: Model 2–4), instead decreased. Compared with children whose mothers had experienced IPV (OR = 1.11; 95% CI: 1.07, 1.15), children whose mothers had experienced all types of violence had a higher risk of stunted (OR = 1.15; 95% CI: 1.07, 1.24).

For wasting, maternal exposure to any form of violence and combinations of all forms of violence were negatively associated with child wasting. Women who were classified as having domestic violence in their relationship were 19% less likely to have children with signs of wasting (OR = 0.81; 95% CI: 0.75, 0.87), and this association was maintained in all single-adjusted models of wasting (Figures 1A–D: Model 1–4). Moreover, IPV in the last 12 months reduced the degree of protection for wasting.

As for underweight, in general, IPV was not significantly associated with underweight. However, after controlling for household-related variables (Figure 1C: Model 3), women who responded to physical and emotional violence were approximately 7% less likely to have an underweight child (OR = 0.93; 95% CI: 0.89, 0.98).

## 4 Discussion

This article focused on the relationship between IPV and the nutritional status of children. We found that IPV was positively associated with child stunting in SSA countries and had a protective effect against child wasting. However, IPV seemed to be not related to child underweight. IPV in the last 12 months significantly increased the likelihood of stunting, and IPV in the last 12 months reduced the degree of protection for wasting. Sexual violence showed a strong negative effect on childhood stunting. This study illustrated that the prevalence of IPV is very high in SSA countries. Moreover, violence is specifically divided into three independent violence and various forms of violence in this study, which is introduced to further display the relationship in detail.

For stunting, physical violence was found to be positively associated with child stunting, and physical violence in the last 12 months increased this association, unexpectedly. Moreover, sexual violence showed a strong positive correlation with childhood stunting, and the correlation did not change after controlling for all predictors, indicating that sexual violence played a large role in childhood stunting. In addition, there was also a significant positive correlation between emotional violence and child stunting. Mothers suffering from IPV will lead to low mothers’ rights and a lack of autonomy in the family. It can easily lead to the decision-making related to the child malnutrition (21, 22). The mother’s experience of violence affects their nutritional status and mental health, and poor mental health in women due to exposure to violence may impair their ability to care for children, including feeding and health-seeking behaviors, both of which affect adverse nutritional outcomes in children (23–25). Moreover, violence can lead to bad living habits, such as alcoholism during pregnancy and maltreatment, which will affect the results of birth and growth, and create a poor growth environment for children (26). In previous studies, numerous results have shown that IPV was associated with adverse pregnancy outcomes in pregnant women, as well as intrauterine growth restriction, postpartum depression, and low birth weight (27–29), which increases the risk of neonatal deaths, for survivors, of stunting by 2 years of age (30).

Wasting was unexpectedly inversely associated with IPV. Related studies have also reported this association (15, 31), but other studies have shown that IPV has an adverse effect on child wasting, which may be due to sample size and sampling errors (32, 33). Wasting and stunting indicate different meanings of malnutrition. Wasting reflects short-term acute malnutrition, while stunting reflects long-term chronic malnutrition, and different periods of violence have different effects on different nutritious statuses. Furthermore, wasting is commonly more prominent at lower age (wasting peaks at 12–23 months) (34), and the larger age may be less prone to wasting, which may have contributed to a lack of association or even inversed association (35). This can be explored by adjusting for age, or differential analysis at lower age and older age.

For underweight, there was no clear correlation between underweight and violence. Although this finding is consistent with some studies (35, 36), other studies show that exposure to IPV increases the probability of underweight (37, 38). For example, a study from India indicates positive associations between past domestic violence and underweight among women and their children. These inconsistent findings may be attributed to the study population and socioeconomic background. It is possible that the intrauterine environment or genetic programming has determined the level of body weight (39). These inconsistent findings require future research to closely explore this issue.

Compared to previous studies, this study expands the scope to study the sub-Saharan demographic and health data. On the basis of large sample size, the data has better representativeness and reliability. It also improves the stability of statistical results and the conclusions are more convincing. We modeled the predictors of outcomes and controlled for the effects of each model to increase the degree of authenticity of the association between exposure and outcomes. We showed geographic and spatial differences in prevalence across regions and countries, providing the basis for focused intervention and equal allocation of substantial resources for public health.

There are several limitations to consider. First, as this was an analysis of secondary data, we were limited to data collected by DHS and could not observe the influence or moderating effect of unmeasured factors. We removed older datasets from the DHS database, perhaps because there were no surveys that included violence modules, or because some sub-Saharan countries did not have child databases, so the results were not fully representative of all sub-Saharan countries and only applied to countries within the scope of the study. However, between 2010 and 2011 this is almost a 12-year gap between the various datasets, which may result in huge discrepancies in the population characteristics, child nutrition landscape, and even IPV situation in various countries. Second, there is bias in this study, most likely the recall bias from the unclear recall of their past exposure history, and the loss of follow-up leading to missing values and incomplete information investigation. Third, further limitations include the inherent causal coexistence of cross-sectional studies. That means the inability to determine the chronological order of disease and certain factors, so only correlation analysis can be performed to provide clues for etiological studies. Finally, due to the lack of standardized measurement scores for IPV, studies cannot explore the linear association between violence strength and child nutritional status.

## 5 Conclusion

IPV is positively associated with child stunting in SSA countries. Sexual violence showed a strong positive correlation with childhood stunting. Wasting was unexpectedly negatively associated with IPV. There was no clear correlation between underweight and violence. Future studies and practice should further focus on underweight. Sexual violence should be more likely to focus on. Further corresponding policy and programmatic implications such as providing special attention and psychological counseling to groups affected by IPV are needed in future research and practice to reduce the occurrence of IPV.

## Data availability statement

Publicly available datasets were analyzed in this study. This data can be found at: https://dhsprogram.com/data/Guide-to-DHS-Statistics/index.cfm.

## Ethics statement

The data for this study was based on the DHS's public survey datasets, and the ethical approval and participant consent were therefore not required for this study. We had granted the right to download and use the data for this study. There were no recorded names or home addresses of individuals in these datasets, and the ethics in its survey are available at: https://dhsprogram.com/Methodology/Protecting-the-Privacy-of-DHS-Survey-Respondents.cfm.

## Author contributions

KL conceptualized and designed the study, carried out the initial analyses, drafted the initial manuscript, and critically reviewed and revised the manuscript. SB, DC, and YuZ collected data. ML, BC, ZZ, YijZ, YingZ, and YJ designed the data collection instruments, collected data, and critically reviewed and revised the manuscript. YX critically reviewed and revised the manuscript. PZ conceptualized and designed the study, coordinated and supervised data collection, and critically reviewed and revised the manuscript for important intellectual content. All authors contributed to the article and approved the submitted version.
