# Supplementary material for: The relationship between intimate partner violence and child malnutrition: a retrospective study in 29 sub-Saharan African countries
Source: Front Public Health. 2024 Jan 5;11:1231913. doi: 10.3389/fpubh.2023.1231913 (PMC10796995; doi:10.3389/fpubh.2023.1231913)
Supplement: Supplementary file 1 [file Table_1.DOCX]

**Supplementary Files**

**Supplementary File 1. Description and analysis coding plan of the selected study variables**

| **Variables** | **Description** | **Analysis Coding** |
| --- | --- | --- |
| Dependent variables |  |  |
| Stunting | Height-for-age (HAZ) index | 0 = Not-stunting (HAZ -2SD and above)  1 = Stunting (HAZ < -2SD) |
| Underweight | Weight-for age (WAZ) index | 0 = Not-underweight (WAZ-2SD and above) 1 = Underweight (WAZ < -2SD) |
| Wasting | Weight-for-height (WHZ) index | 0 = Not-wasted (WHZ-2SD and above)  1 = Wasted (WHZ < -2SD) |
| Independent variables |  |  |
| Physical violence | Ever been pushed, shook, or had something thrown by the husband/partner; ever been slapped by the husband/partner; ever been punched with a fist or hit by something harmful by the husband/partner; ever been kicked or dragged by the husband/partner; ever been strangled or burnt by the husband/partner; ever being threatened with knife/gun or other weapons by the husband/partner; ever experienced CS physical violence by the husband/partner; ever had arm twisted or hair pulled by the husband/partner | 0 = Not any physical violence by partner  1 = Any physical violence by partner |
| Sexual violence | Ever been physically forced into unwanted sex by husband/partner, forced into other unwanted sexual acts by husband/partner, or physically forced to perform sexual acts respondent didn't want to | 0 = Not any sexual violence by partner  1 = Any sexual violence by partner |
| Emotional violence | Ever being humiliated by the husband/partner, threatened with harm by the husband/partner, insulted, or made to feel bad by the husband/partner | 0 = Not any emotional violence by partner  1 = Any emotional violence by partner |
| Physical violence (12m) | Ever exposed to any forms of physical violence in the past 12 months | 0 = Not any physical violence by partner in the past 12 months  1 = Any physical violence by partner in the past 12 months |
| Sexual violence (12m) | Ever exposed to any forms of sexual violence in the past 12 months | 0 = Not any sexual violence by partner in the past 12 months  1 = Any physical sexual by partner in the past 12 months |
| Emotional violence (12m) | Ever exposed to any forms of emotional violence in the past 12 months | 0 = Not any emotional violence by partner in the past 12 months  1 = Any emotional violence by partner in the past 12 months |
| Covariates |  |  |
| Child’s gender | Sex of children | 1 = Male  2 = Female |
| Child’s age in months | Current age of the child（months） | 1 = < 12months 2 = 12–35months 3 = 36–59months |
| Duration of breast-feeding | Breastfeeding status | 93 = Ever breastfeeding  94 = Never breastfeed  95 = Still breastfeeding |
| Complementary feeding | Gave child eggs; Gave child meat (beef, pork, lamb, chicken, etc); Gave child pumpkin, carrots, squash (yellow or orange inside); Gave child any dark green leafy vegetables; Gave child liver, heart, other organs; Gave child fish or shellfish (in the 6 months) | 0 = No  1 = Yes |
| Vitamin A in last 6 months | Vitamin A supplements in the six months preceding the interview | 0 = No  1 = Yes |
| Wealth index | Wealth quintiles | 1 = Poorest  2 = Poorer  3 = Middle  4 = Richer  5 = Richest |
| Type of place of residence | Urban or rural | 1 = Urban  2 = Rural |
| Maternal education | Educational attainment | 0 = No education  1 = Incomplete primary  2 = Complete primary  3 = Incomplete secondary  4 = Complete secondary  5 = Higher |
| Marital status | Current marital status | 0 = Never in union  1 = Married  2 = Living with partner  3 = Widowed  4 = Divorced  5 = No longer living together/separated |
| Mother’s BMI | Body Mass Index (calculated using measured height and weight) | 1 = Underweight (<18.5 kg/m^2^) 2 = Normal (18.5–24.9 kg/m^2^) 3 = Overweight (25.0–29.9 kg/m^2^) 4 = Obese (≥ 30 kg/m^2^) |
| Mother’s hemoglobin level | Hemoglobin level adjusted for altitude and smoking (g/dl - 1 decimal) | 1 = Severe anemia(<80g/dl)  2 = Moderate anemia(80-110g/dl)  3 = Mild anemia(110-120g/dl)  4 = No anemia(>120g/dl) |

**Research Checklist--**[***STROBE guidelines***](http://www.equator-network.org/reporting-guidelines/the-strengthening-the-reporting-of-observational-studies-in-epidemiology-strobe-statement-guidelines-for-reporting-observational-studies/)

| Section/topic | Item number | Recommendation |
| --- | --- | --- |
| Title and Abstract | 1 | We collected and analyzed 29 SSA countries’ Demographic and Health Surveys data (2010-2021). An adjusted binary logistic regression model was used to test the relationship between maternal IPV experience and children's nutritional status (stunting, underweight, wasting). Our findings showed that IPV was positively associated with child stunting in SSA countries. Sexual violence showed a strong positive correlation with childhood stunting. Wasting was unexpectedly negatively associated with IPV. There was no clear correlation between underweight and IPV. |
| Introduction |  |  |
| Background/rationale | 2 | Intimate partner violence (IPV) and child malnutrition are worldwide public health issues. Assessing the association between IPV and child malnutrition can provide significant global health solutions. |
| Objectives | 3 | This cross-sectional study aims to explore the associations of exposure to the various types of IPV with childhood malnutrition status. |
| Methods |  |  |
| Study design | 4 | We collected and analyzed 29 SSA countries’ Demographic and Health Surveys data (2010-2021). The main exposure variables were various types of IPV, which were classified as physical violence, sexual violence, and emotional violence. The outcome event was the child's development index, which can be roughly divided into stunting, wasting, and underweight. And several covariates, such as child’s gender, child’s age in months, duration of breast-feeding, complementary feeding, and vitamin A in last 6 months classified as child’s factors, wealth index, and type of place of residence classified as household factors, maternal education, marital status, maternal BMI and hemoglobin level classified as maternal-related factors, may have influenced the results. This study analyzed secondary data from the DHS. In this study, we combined the individual and children recode datasets. Women aged 15 to 49 who responded to the violence module were considered. Additionally, the nutritional status of their children in these households were also assessed, based on weight and height measurements. |
| Setting | 5 | This study analyzed secondary data from the DHS. DHS sample designs were two-stage probability samples drawn from an existing sample frame, generally the most recent census frame. Each country’s survey consisted of different datasets including men, women, children, birth, and household datasets. These latest datasets collected in 29 SSA countries from 2010 to 2021 were appended together to investigate the association between the nutritional status of children under 5 years old and maternal IPV. |
| Participants | 6 | Women aged 15 to 49 who responded to the violence module were considered. Additionally, the nutritional status of their children in these households were also assessed. 186,138 children under 5 years old were included in the analysis. |
| Variables | 7 | Using WHO growth criteria as a reference, stunting, underweight, and wasting were defined as Z-scores less than -2 standard deviations (SD) from the median height-for-age (HAZ), weight-for-age (WAZ), and weight-for-height (WHZ), respectively. In other words, “stunting” means HAZ < -2 and “no stunting” means HAZ >= -2, “underweight” means WAZ < -2 and “no underweight” means WAZ >= -2, “wasting” means WHZ < -2 and “no wasting” means WHZ >= -2. The main exposure variables were types of IPV, which were classified as physical violence, sexual violence, and emotional violence. We also created the presence or absence of IPV in the past 12 months by time. Several covariates, such as child’s gender, child’s age in months, duration of breast-feeding, complementary feeding, and vitamin A in last 6 months classified as child’s factors, wealth index, and type of place of residence classified as household factors, maternal education, marital status, maternal BMI and hemoglobin level classified as maternal-related factors, may have influenced the results. Factors that have been reported in the literature were identified as covariates for assessing child nutritional status include the child's age in months, child’s gender, number of birth orders, birth weight, diarrhea in the past two weeks, mother's age group, mother's highest level of education, place of residence, mother's BMI, wealth quintile. These factors are associated with stunting, underweight, and wasting conditions in children under 5 years of age. Therefore, relevant confounding factors were considered in this study and corrected to obtain more reliable results. |
| Data sources/measurement | 8 | The coding plan of the selected study variables is shown in Supplementary File 1. |
| Bias | 9 | As this was an analysis of secondary data, we were limited to information collected by DHS and could not observe the influence or moderating effect of unmeasured factors. We removed older datasets from the DHS database, perhaps because there were no surveys that included violence modules, or because some sub-Saharan countries did not have child databases, so the results were not fully representative of all sub-Saharan countries, and only applies to countries within the scope of the study. And most likely the recall bias from the unclear recall of their past exposure history, and the loss of follow-up leading to missing values and incomplete information investigation. |
| Study size | 10 | These latest datasets from DHS collected in 29 SSA countries from 2010 to 2021 were appended together. 186,138 children under 5 years old were included in the analysis by removing missing data. |
| Quantitative variables | 11 | In 29 Sub-Saharan countries, we described the incidence of the nutritional status of children by countries and regions (East Africa, Southern Africa, West Africa, Central Africa), which provides a picture of their prevalence. |
| Statistical methods | 12 | IPV and child nutrition status were statistically described for each country to reveal the prevalence.  The differences between the incidence of outcome events caused by various independent variables and covariates were tested by Pearson's Chi-Square test. An adjusted binary logistic regression model was used to examine the relationship between maternal IPV experience and children's nutritional status (stunting, underweight, wasting). Four weighted multivariable logistic regression models for each measure of child undernutrition were used to correct the odds ratio (OR). The first model controlled for child-related factors, namely child’s gender, child’s age in months, duration of breastfeeding, complementary feeding and vitamin A in the last 6 months. The second model controlled for variables related to household factors: metropolitan status, and the household wealth index. The third model controlled for maternal-related factors: maternal education, marital status, BMI, and hemoglobin level. The final model included all variables representing the three domains (child, maternal, and household factors) and measures of IPV. This approach tested the impact of IPV on child malnutrition after controlling for similar factors. The regression results were presented as the estimated adjusted odds ratio (AOR) with 95% confidence intervals (CI). The significance level of regression analysis was set at P <0.05. All statistical analyses were performed using STATA version 15.1. |
| Results |  |  |
| Participants | 13 | 186,138 children under 5 years old were included in the analysis by removing missing data. |
| Descriptive data | 14 | In the study, a total of 186,138 children under the age of five were included, of which 80,864 (43.4%) were from East Africa, 2,857 (1.5%) from Southern Africa, 70,216 (37.7%) from West Africa and 32,201 (17.3%) from Central Africa. Nearly 40.0% of the women had no education at all, and only 3.0% had college or above education. 128,268 (68.9%) of the subjects resided in rural areas, and about 47.1% of them lived in poverty. Among the children, 93,828 (50.4%) were boys, 39,894 (21.4%) were in infancy (0-11months), 75,450 (40.5%) were in toddler (12-35months), and 70,794 (38.0%) were in pre-school age (36-59months). 28.6% of women had experienced physical violence in their lives. 10.0% of women had been subjected to sexual violence by their partners, and 28.5% of women suffered from emotional violence. 5.8% of women had experienced all three types of violence, and 40.3% had experienced any type of violence. |
| Outcome data | 15 | In East Africa, the prevalence of stunting is 27.8%. Besides, the prevalence of stunting is 19.0% in Southern Africa and 23.8 % in West Africa, with Central Africa having the highest incidence of stunting at 33.2%. Central Africa also had the highest incidence of underweight (25.9%). The prevalence of stunting is 47.4%, underweight rate is 35.1% in Burundi, and this is the highest among countries in the research. The incidence of wasting is relatively low. More importantly, 50,113 (27.1%) of the children remained stunted, 11,329 (6.1%) remained wasted, 39,459 (21.3%) remained underweight in all regions. 10.0% of women had been subjected to sexual violence by their partners, and 28.5% of women suffered from emotional violence. 5.8% of women had experienced all three types of violence, and 40.3% had experienced any type of violence. |
| Main results | 16 | For stunting, in the crude model (Model 1), women who were identified as having experienced any physical violence were 8.0% more likely to have a stunted child than women who had not experienced physical violence (OR = 1.08, 95% CI: 1.03, 1.12). After controlling for differences in child characteristics (Model 2), this association remained (AOR = 1.07, 95% CI :1.01, 1.13). Mothers who had experienced sexual violence had a 21.0% greater likelihood of having a child with developmental delay (OR = 1.21, 95% CI:1.14, 1.28), which remained statistically significant after controlling for relevant factors or even all variables (AOR = 1.11, 95% CI:1.02, 1.21).  For wasting, maternal exposure to any forms of violence and combinations of all forms of violence were negatively associated with child wasting. Women who were classified as having domestic violence in their relationship were 19% less likely to have children with signs of wasting (OR = 0.81, 95% CI: 0.75, 0.87), this association was maintained in all single adjusted models of wasting (Model 1-4).  As for underweight, in general, IPV was not significantly associated with underweight. |
| Other analyses | 17 | IPV in the last 12 months significantly increased the likelihood of stunting, and IPV in the last 12 months reduced the degree of protection for wasting. |
| Discussion |  |  |
| Key results | 18 | We found that IPV had a detrimental effect on child stunting and a protective effect on child wasting. However, IPV seemed to be not related to child underweight. Sexual violence showed a strong negative effect on childhood stunting. |
| Limitations | 19 | First, as this was an analysis of secondary data, we were limited to information collected by DHS and could not observe the influence or moderating effect of unmeasured factors. We removed older datasets from the DHS database, perhaps because there were no surveys that included violence modules, or because some sub-Saharan countries did not have child databases, so the results were not fully representative of all sub-Saharan countries, and only applies to countries within the scope of the study. There is bias in this study, most likely the recall bias from the unclear recall of their past exposure history, and the loss of follow-up leading to missing values and incomplete information investigation. Second, further limitations include the inherent causal coexistence of cross-sectional studies. That means the inability to determine the chronological order of disease and certain factors, so only correlation analysis can be performed to provide clues for etiological studies. Finally, due to the lack of standardized measurement scores for IPV, studies cannot explore the linear association between violence strength and child nutritional status. |
| Interpretation | 20 | Mothers suffering from IPV will lead to low mothers' rights and a lack of autonomy in the family, which can easily affect the decision-making related to the growth and development of children. The mother’s experience of violence affects their nutritional status and mental health, and poor mental health in women due to exposure to violence may impair their ability to care for children, including feeding and health-seeking behaviors, both of which affect adverse nutritional outcomes in children. Moreover, violence can lead to bad living habits, such as alcoholism during pregnancy and maltreated, which will affect the results of birth and growth, and create a poor growth environment for children. numerous results have shown that IPV was associated with adverse pregnancy outcomes in pregnant women, as well as intrauterine growth restriction, postpartum depression, and low birth weight, which increases the risk of neonatal deaths and, for survivors, of stunting by 2 years of age. |
| Generalizability | 21 | This study expands the scope to study sub-Saharan demographic and health data. On the base of a large sample size, it will have sufficient power to detect the true effect of independent variables, making the results generalizable. |
| Other information |  |  |
| Funding | 22 | This study was financially supported by National Key Research and Development Program of China (2021YFC2700700 and 2021YFC2700705), Peking University Third Hospital Incubation Fund for Youth (No. BYSYFY2021014), Peking University Third Hospital Clinical Queue Construction Project（No. BYSYDL2022008） |
